# Evaluation of neurological disorders that develop concurrently with COVID-19 pneumonia: a retrospective analysis

**DOI:** 10.1590/0004-282X-ANP-2021-0059

**Published:** 2022-04-20

**Authors:** Irem TASCI, Ferhat BALGETIR, Bulent MUNGEN, Caner Feyzi DEMIR, Murat GONEN, Leman Acun DELEN, Osman KURT

**Affiliations:** 1 Malatya Turgut Ozal University, Medicine Faculty, Department of Neurology, Malatya, Turkey.; 2 Firat University, Medicine Faculty, Department of Neurology, Elazig, Turkey.; 3 Malatya Training Research Hospital, Anesthesia Clinic, Malatya, Turkey.; 4 Firat University, Medicine Faculty, Department of Public Health, Elazig, Turkey.

**Keywords:** COVID-19, Neurology, Referral and Consultation, Central Nervous System Diseases, Peripheral Nervous System Diseases, COVID-19, Neurologia, Encaminhamento e Consulta, Doenças do Sistema Nervoso Central, Doenças do Sistema Nervoso Periférico

## Abstract

Background: During the pandemic, many neurological symptoms have been evaluated as complications of COVID-19 pneumonia. Objective: To investigate the frequency and characteristics of neurological findings, and their effects on the prognosis of patients with COVID-19 pneumonia who consulted with the Neurology department. Methods: Data on 2329 patients who were hospitalized with the diagnosis of COVID-19 pneumonia in our hospital were scanned. The clinical, laboratory and radiological findings relating to treatment of 154 patients who required neurological consultation were retrospectively evaluated by reviewing the clinical notes. Results: The number of COVID-19 pneumonia patients who required neurological consultations while hospitalized in the ICU was 94 (61.0%). The most common symptom among these patients was hyperactive delirium. Mean age, ferritin levels and CRP values ​​of those with delirium were higher, while the mean lymphocyte percentage were lower, than those of the patients without delirium. Epileptic seizures were observed in eight patients without an epilepsy diagnosis. Two patients were diagnosed with GBS and one patient with ICU neuropathy. The D-dimer levels of patients with acute hemorrhagic CVD and the thrombocyte levels of patients with acute ischemic CVD were found to be higher than in patients without acute ischemic CVD. Conclusion: The proportion of patients who required neurological consultations was higher in the ICUs. We observed neurological symptoms more frequently in the advanced age group. There were no significant increases in the incidence of other neurological conditions except delirium, in COVID-19 patients. We think that further studies are needed to support our data.

## RESUMO

Antecedentes: Durante a pandemia, muitos sintomas neurológicos foram avaliados como complicações da pneumonia por COVID-19. Objetivo: Investigar a frequência e as características dos achados neurológicos e seus efeitos no prognóstico de pacientes com pneumonia por COVID-19 que consultaram o departamento de Neurologia. Métodos: Foram analisados os dados de 2.329 pacientes internados com diagnóstico de pneumonia por COVID-19 em nosso hospital. Os achados clínicos, laboratoriais e radiológicos relativos ao tratamento de 154 pacientes que necessitaram de consulta neurológica foram avaliados retrospectivamente por meio da revisão das anotações clínicas. Resultados: O número de pacientes com pneumonia por COVID-19 que necessitaram de consultas neurológicas enquanto internados na UTI foi de 94 (61,0%). O sintoma mais comum entre esses pacientes foi o delírio hiperativo. A média de idade, os níveis de ferritina e os valores de PCR daqueles apresentando delírios foram maiores, enquanto a porcentagem média de linfócitos foi menor do que em pacientes sem delírios. Crises epilépticas foram observadas em oito pacientes sem diagnóstico de epilepsia. Dois pacientes foram diagnosticados com SGB e um paciente com neuropatia na UTI. Os níveis de dímero D de pacientes com DCV hemorrágica aguda e os níveis de trombócitos de pacientes com DCV isquêmica aguda foram maiores do que em pacientes sem DCV isquêmica aguda. Conclusão: A proporção de pacientes que necessitaram consultas neurológicas foi maior nas UTIs. Observamos sintomas neurológicos com mais frequência em pacientes de faixa etária avançada. Não houve aumentos significativos na incidência de outras condições neurológicas, exceto delírio, em pacientes com COVID-19. Acreditamos que mais estudos são necessários para apoiar nossos dados.

## INTRODUCTION

The World Health Organization (WHO) declared that COVID-19 constituted a pandemic on March 11, 2020^1^. According to WHO data, the virus had infected 172,956,039 people worldwide as of June 7, 2021, and had caused 3,726,466 deaths^2^.

Two coronaviruses previously identified as SARS-CoV-1 and MERS-CoV have caused large-scale epidemics^3^ ^,^ ^4^. SARS-CoV-2 may have higher neuroinvasive potential than previous coronaviruses^5^.

Viruses enter the central nervous system (CNS) essentially through hematogenous and neuronal retrograde propagation pathways. SARS-CoV-2 can also bind to angiotensin receptor 2 (ACE2), which is expressed in the capillary endothelium of the blood brain barrier (BBB), to access the CNS^6^ ^,^ ^7^. The ACE2 receptor is expressed intensely in the cerebellum, thalamic nuclei, inferior olivary nucleus, ventrolateral medulla and tractus solitarius nucleus, in the CNS^1^ ^,^ ^8^. SARS-CoV-2 has higher affinity for the ACE2 receptors found in neurons and endothelial cells than does SARS-CoV-1^7^.

The mechanisms that have been suggested for the development of various neurological syndromes include direct viral neuronal damage, a hyperinflammatory syndrome secondary to viremia, para-infectious and post-infectious inflammatory or immune-mediated disorders, sepsis, hyperpyrexia, hypoxia, hypercoagulopathy and critical illness. Several neurological conditions, including encephalopathy, meningoencephalitis, ischemic stroke, acute necrotizing encephalopathy and Guillain-Barré syndrome (GBS), have been found to coexist with COVID-19^9^.

Considering the high rates of COVID-19 infection in the general population, it is important to distinguish whether COVID-19 is associated with neurological involvement or whether coexistence of neurological diseases is coincidental, with support from scientific data^10^.

In our study, we retrospectively reviewed the files of 2,329 patients who had been diagnosed with COVID-19 pneumonia and hospitalized in the wards or intensive care units (ICUs) of our institution. We conducted statistical analyses on the data regarding neurological findings, demographic data, relationships with other chronic diseases, radiology and laboratory findings and drug use, with regard to 154 patients who required neurological consultations (NC).

## METHODS

The files of 2,329 patients with a diagnosis of COVID-19 pneumonia who were hospitalized in the wards and ICUs of our institution, between March 23, 2020, and October 1, 2020, were examined. The clinical findings, radiological findings and treatment records of 154 patients who required NC due to neurological symptoms were screened retrospectively by evaluating the consultation notes and patient files. The reason for requesting NC, neurological diagnoses, neurological examination findings and radiological data of all patients were examined. Patients under 18 years of age, those with a negative PCR test and/or without findings compatible with COVID-19 pneumonia on chest computed tomography imaging were not included in the evaluation.

The study protocol was approved by the Republic of Turkey Ministry of Health Scientific Research Platform and by Firat University Medical School Clinical Research Ethics Board.

The age, sex, ICU admission and length of hospitalization of the patients who required NC were evaluated. The PCO_2_, pH, PaO_2_/FiO_2_ ratio, positive end expiratory pressure (PEEP), oxygen saturation, lymphocyte, white blood cell (WBC), thrombocyte, ferritin, D-dimer, pro-brain natriuretic peptide (pro-BNP), fibrinogen and C-reactive protein (CRP) values of the patients were recorded. The treatments that they received were recorded. The frequencies of neurological or non-neurological chronic comorbid diseases among the patients in this group were examined. The neurological findings magnetic resonance imaging (MRI), CT, electroencephalography (EEG) and electromyography (EMG) findings of the patients were evaluated. The demographic data, risk factors, treatments and radiological and laboratory findings of patients who were diagnosed with delirium, acute ischemic or hemorrhagic stroke were evaluated. Delirium patients were identified by using the Richmond Agitation Sedation Scale (RASS). RASS is a 10-point scale, where (0) indicates a calm and alert state, while the levels from +1 to +4 indicate increasing levels of agitation and the levels from -1 to -5 indicate increasing levels of sedation^11^. It has been stated that patients with RASS scores between -4 and +4 can be evaluated as having delirium^12^.

### Statistical analysis

The data were analyzed by using the Statistical Package for the Social Sciences (SPSS) v.22 software (SPSS Inc., Chicago, IL, USA) Descriptive statistics were expressed as the number and percentage for categorical data and as the mean±standard deviation for continuous data. Pearson chi-square analysis was used to compare categorical variables between groups. The Kolmogorov-Smirnov test was used to evaluate the normality of distribution of continuous variables. The one-way ANOVA test was used to compare the normally distributed numerical measurements in more than two groups. Student’s *t*-test was used for comparison of paired groups. P<0.05 were accepted as statistically significant.

## RESULTS

The files of a total of 2,329 COVID-19 pneumonia patients were screened in this study. The number of patients who required NC was 154. NC were requested for 94 patients (61.0% of the patients with COVID-19 pneumonia seeking NC) and were not requested for 486 patients (22.3% of all COVID-19 pneumonia patients) who were hospitalized in the ICU (p<0.001). The proportion of males among the patients who required NC was 64.3% and it was 56.5% among who did not require NC (p=0.058). The mean age of the patients who required NC (72.3±15.0 years) was found to be significantly higher than the mean age of the patients who did not require consultations (64.4±17.1 years) (p<0.001). The mean age was 72.3±15.0 years (range: 21-96) and 99 patients (64.3%) were male. The most commonly used antiviral medication among the patients who required NC was favipiravir (67.5%). At least one antibiotherapy agent was given to 97.6% of the patients, anticoagulants to 90.3% and steroid medication to 63.6%. While 36.4% of the patients who required NC were intubated, 33.8% died. The demographic characteristics and disease related features of the patients are given in Table 1.

**Table 1.** Demographic characteristics and disease-related features of the patients.

		Number	%		Mean±SD
Sex	Male	99	64.3	Age	72.3±15.0
	Female	55	35.7	Hospital stay (days)	12.8±11.3
				Ph	7.4±0.1
Hospitalization	Ward	60	39.0	PCO_2_	39.3±10.9
	ICU	94	61.0	Lymphocyte (%)	16.7±25.7
Intubated		56	36.4	WBC	11.6±7.8
Exitus		52	33.8	Thrombocyte	217.1±103.9
HT		79	51.6	Ferritin	552.7±564.1
LF		2	1.3	D-dimer	2.8±3.6
CRF		8	5.2	Pro-BNP	5358.9±9102.3
DM		39	25.3	Fibrinogen	510.2±212.2
DVT		1	2.6	CRP	9.3±7.7
Delirium		95	61.7		
COPD		24	15.6		
Cancer		4	2.6		
CF		28	18.2		
AF		8	17.4		
CAD		57	37.0		
Acute hemorrhagic CVD		6	3.9		
Chronic hemorrhagic CVD		10	6.5		
Acute ischemic CVD		26	16.25		
Acute SVT		1	2.6		
Chronic ischemic CVD		57	37.0		
Parkinson’s disease		10	6.5		
Epilepsy		5	3.2		
Dementia		43	27.9		
Guillain-Barre syndrome		2	1.9		
Critical disease neuropathy		1	2.6		
Moxifloxacin		46	74.2		
Azithromycin		36	23.4		
Tocilizumab		2	1.3		
Ritonavir+Lopinavir		6	3.9		
Oseltamivir		20	13.0		
Favipiravir		104	67.5		
Enoxaparin		139	90.3		
Deltacortril		98	63.6		
Chloroquine		89	57.8		

ICU: intensive care unit; HT: hypertension; LF: liver failure; CRF: chronic renal failure; DM: diabetes mellitus; DVT: deep vein thrombosis; COPD: chronic obstructive pulmonary disease; CF: cardiac failure; AF: atrial fibrillation; CAD: coronary artery disease; CVD: cerebrovascular disease; SVT: sinus vein thrombosis.

**Figure 1.**
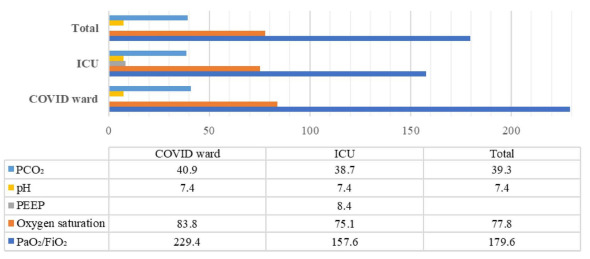
Comparison of respiratory parameters of patients in COVID wards and intensive care units. The PaO_2_/FiO_2_ ratio and O_2_ saturation values of the patients hospitalized in the COVID ward were found to be significantly higher than the values of the patients hospitalized in the intensive care unit (p<0.001). However, no significant difference was observed between the COVID ward and ICU, regarding PCO_2_ (p=0.317) and pH (p=0.691) values. The mean PEEP value of intensive care patients was 8.4±2.0.

ICU: intensive care unit; PEEP: positive end expiratory pressure.

In comparing respiratory parameters of the COVID-19 patients between those hospitalized in the wards and those in the ICUs, it was found that the PaO_2_/FiO_2_ ratios and oxygen saturation levels of the patients hospitalized in the wards were significantly higher (p<0.001). However, no statistically significant difference was found between pH and PCO_2_ parameters. It was found that the mean PEEP value of the patients hospitalized in the ICU was 8.4±2.0 (Figure 1).

It was observed that the most common neurological symptom among the patients was hyperactive delirium. The most common findings on both MRI and CT were cerebrocerebellar atrophy and presence of common ischemic gliotic areas. The other most common findings, following atrophy and chronic ischemic gliotic areas in CT, were chronic infarction (17.6%) and acute infarction (8.4%). In addition, intracerebral hemorrhage was observed in six patients (4.6%). EMG was performed on eight patients with complaints of paraparesis, tetraparesis and hypoesthesia. The EMG findings included diffuse sensorimotor polyneuropathy compatible with ICU neuropathy in one patient, polyneuropathy with acute axonal damage in one patient and EMG findings compatible with acute demyelinating-type sensorimotor polyneuropathy in one patient. EEG examinations were performed on four patients presenting confusion who were hospitalized in wards. In two patients, a slowdown in the background rhythm was observed in delta activity, whereas the EEG was normal in two patients (Table 2).

**Table 2.** Neurological symptoms and magnetic resonance imaging, computed tomography and electromyography findings of the patients.

	Number	%
Neurological symptoms		
Paraparesis and tetraparesis	3	1.9
Hyperactive delirium*	82	52.9
Epileptic seizure	13	8.4
Hypoactive delirium* (encephalopathy such as lethargy, stupor or confusion)	23	14.9
Coma	6	3.88
Acute focal neurological deficit (hemiparesis, aphasia, etc.)	24	15.6
Headache	9	5.8
Dizziness	9	5.8
Syncope	4	2.6
Anisocoria	2	1.3
MRI findings		
Acute infarction bilaterally in hemispheres	3	4.2
Acute infarction in the left hemisphere	12	16.7
Acute infarction in the right hemisphere	7	9.7
Chronic infarction	10	13.9
Thrombus in the right transverse sinus	1	1.4
Hemorrhagic infarction in the right hemisphere	1	1.4
Cerebrocerebellar atrophy or common ischemic gliotic areas	29	40.3
Other (craniectomy defect or intracranial mass)	4	5.5
CT findings		
Hematoma in the brain stem and cerebellum	2	1.5
Hematoma in the right hemisphere and subarachnoid hemorrhage	4	3.1
Acute infarction	11	8.4
Chronic infarction	23	17.6
Cerebrocerebellar atrophy or common ischemic gliotic areas	66	50.4
Other (hydrocephalus, craniectomy defect, intracranial mass, calcification or vascular occlusion)	10	7.6
EMG findings		
Diffuse sensorimotor polyneuropathy	1	12.5
Polyneuropathy with acute axonal damage	1	12.5
Acute demyelinating-type polyneuropathy	1	12.5
Normal	5	62.5
EEG findings		
Diffuse deceleration in delta frequency	2	50
Normal	2	50

MRI: magnetic resonance imaging; CT: computed tomography; EEG: electroencephalography; EMG: electromyography. *The data on mixed-type delirium patients are included in this group.

**Figure 2.**
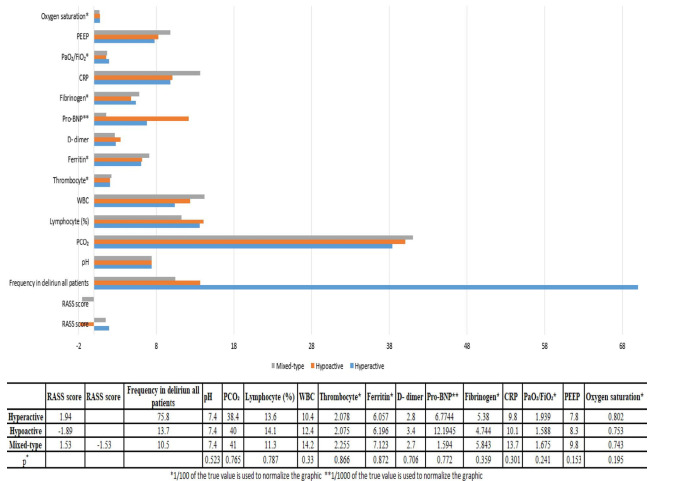
Richmond Agitation Sedation Scale scores and respiratory, infectious and coagulation parameters in delirium subtypes. When the patients who required neurology consultations were evaluated according to the subtypes of delirium, it was found that hyperactive delirium was present in 75.8%, hypoactive delirium in 13.7% and mixed-type delirium in 10.5% of the patients. There were no statistically significant differences between the subtypes of delirium in terms of RASS scores. There were no significant differences between delirium subgroups in terms of respiratory, infectious or coagulation data, as indicated in the table above (p>0.05).

Delirium was detected in 61.7% of the patients who required NC. When the patients who required NC were evaluated according to the subtypes of delirium, it was found that hyperactive delirium was present in 75.8%, hypoactive delirium in 13.7% and mixed- type delirium in 10.5% of all the delirium patients. The RASS scores of the patients were found to be 1.94±0.73 in hyperactive delirium, -1.89±0.77 in hypoactive delirium and 1.53±0.45 and -1.53±0.45 in mixed-type delirium, respectively. There were no significant differences between the delirium subtypes in terms of RASS scores. In addition, there were no significant differences between the delirium subtypes in terms of pH, PCO_2_, PaO_2_/FiO_2_ ratio, PEEP, oxygen saturation, WBC, lymphocyte, thrombocyte, CRP, fibrinogen, ferritin, D-dimer and pro-BNP levels (Figure 2).

RASS: Richmond Agitation Sedation Scale.

**Figure 3.**
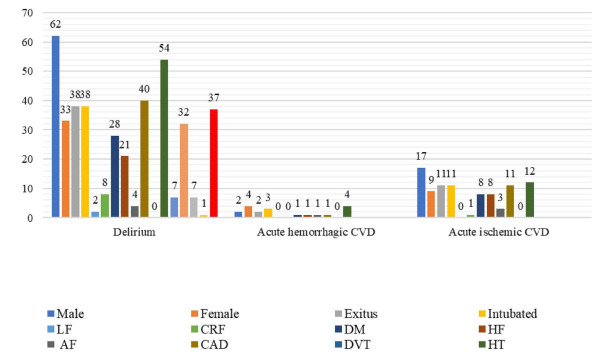
Incidence of chronic diseases among patients with delirium, acute ischemic cerebrovascular disease and acute hemorrhagic cerebrovascular disease. The rate of delirium was found to be significantly higher among patients who died and those with chronic renal failure (p=0.024) and dementia (p<0.001). The rate of delirium was found to be significantly lower among patients with acute cerebrovascular disease (p<0.001) and those with a previous diagnosis of cerebrovascular disease (p=0.012). No statistically significant association was observed between acute ischemic and hemorrhagic cerebrovascular disease patients and other chronic diseases (p>0.05).

LF: liver failure; AF: atrial fibrillation; CRF: chronic renal failure; CAD: coronary artery disease; CVD: cerebrovascular disease; DM: diabetes mellitus; DVT: deep vein thrombosis; HT: hypertension; HF: heart failure.

The delirium rate was found to be significantly higher among patients who died and those with CRF and dementia. This rate was found to be significantly lower among those with acute CVD and those with a previous diagnosis of CVD (Figure 3).

**Figure 4.**
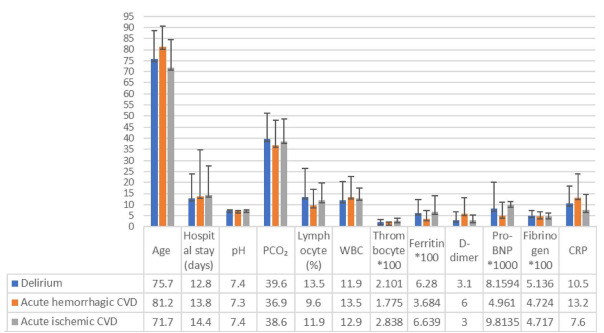
Age, length of hospital stay and laboratory parameters among patients with delirium, acute ischemic cerebrovascular disease and acute hemorrhagic cerebrovascular disease. The average age (p<0.001), ferritin (p=0.04) and C-reactive protein (p=0.015) were significantly higher among patients with delirium than among those without delirium, whereas lymphocyte values (p=0.048) were found to be significantly lower among those with delirium. The D-dimer levels (p=0.03) in patients with acute hemorrhagic cerebrovascular disease were found to be significantly higher than the levels in patients without acute hemorrhagic cerebrovascular disease. The thrombocyte levels (p<0.001) in patients with acute ischemic cerebrovascular disease were found to be significantly higher than the levels in patients without acute ischemic cerebrovascular disease.

The average age and the ferritin and CRP levels were significantly higher among patients with delirium than among those without delirium, whereas the lymphocyte percentage was found to be significantly lower in those with delirium (Figure 4).

CRP: C-reactive protein.

The D-dimer levels of patients with acute hemorrhagic CVD were found to be significantly higher than those of patients without this (p=0.03) (Figure 4).

The thrombocyte levels of patients with acute ischemic CVD were found to be significantly higher than those of patients without this (p<0.001) (Figure 4).

## DISCUSSION

In the literature there are large numbers of meta-analyses, reviews and case reports on neurological involvement in patients with COVID-19. In this study we aimed to identify accompanying neurological diseases depending on clinical findings, radiological images and laboratory data.

Helms et al. found that the mean age of COVID-19 patients with neurological symptoms who were hospitalized in the ICU was 62 years, and that 75% of them were male. They reported that 82% of the patients hospitalized in the ICU had neurological symptoms and that the most common symptom was delirium^13^. In our study, the mean age of the patients who required NC was 72 years, and 64% of the patients were male. In this study, we evaluated the COVID-19 patients hospitalized in both the wards and the ICU. The proportion of the patients who required NC was 61% in the ICU and 39% in the wards.

In a study evaluating COVID-19 patients hospitalized in the ICU, it was observed that the most common comorbidities were cardiovascular diseases, respiratory diseases and DM among patients with neurological symptoms^13^. In our study, the most common chronic diseases among patients who required NC were HT (51.6%), previous ischemic stroke (37%), coronary artery disease (37%), dementia (27.9%) and DM (25.3%), respectively. While 36.4% of the patients who required NC were intubated, 33.8% died. In the study by Mao et al. ^14^, it was reported that the rate of developing neurological symptoms was higher among patients with severe disease, as in our study data.

It has become known that headache and dizziness are common among COVID-19 patients. The frequency of headache in COVID-19 was found between 3 and 12.1% in one study, while it was 27% in another study^15^ ^,^ ^16^. In a study, it was reported that dizziness was observed in 8% of COVID-19 patients^16^. In our study, nine patients (5.8%) with resistant headache and nine patients (5.8%) with persistent dizziness had consultations with the Neurology department.

Epileptic seizures developed in 13 patients (0.5% of all patients and 8.4% of the patients who required NC). Three of these patients had common myoclonic seizures that developed after cardiopulmonary resuscitation and the other patients had generalized tonic-clonic seizures (GTCS). Five of these 13 patients had been diagnosed with epilepsy, and all the epileptic patients had GTCS, while eight patients had epileptic seizures for the first time. Three of patients who had GTCS without a diagnosis of epilepsy had acidosis in blood gas analysis, fever, electrolyte disturbance and severe hypoxia that triggered seizures. No emergency pathological condition was detected through brain CT and diffusion MRI. These seizures were considered to be provoked seizures. No antiepileptic treatment was started. Antiepileptic treatment was started in all patients except the patients who had got provoked seizures. EEG was recommended after COVID-19 treatment. A very small number of retrospective studies have reported seizures in COVID-19, with incidence ranging from 0.5 to 1.4%. All types of seizures have been reported in COVID-19 patients^17^.

Brain and/or diffusion MRI was performed on 66 patients, and brain CT was performed on 116 patients who required NC. The most common radiological findings were cerebrocerebellar atrophy and diffuse ischemic gliotic areas, which were detected in 40.3% of MRI and 50.4% of CT examinations. Consistently, Helmes et al. and Kandemir et al. reported that the most common CNS imaging finding in COVID-19 patients was bilateral signal changes in FLAIR in MRI^13^ ^,^ ^18^.

EMG was performed on eight patients. Acute-onset ascending paraparesis was observed in one patient, tetraparesis in two patients, and hypoesthesia in five patients. The complaints of the patient with acute ascending paraparesis developed 15 days after the diagnosis of COVID-19. In the EMG, acute demyelinating-type sensorimotor polyneuropathy without f response was detected. The first patient with tetraparesis developed tetraparesis on the ninth day of hospitalization, due to COVID-19. In the EMG, motor neuropathy with acute axonal damage without f response was observed. The second patient was intubated for 34 days due to COVID-19 and was evaluated because of tetraparesis after being extubated. In the EMG, subacute-period diffuse sensorimotor polyneuropathy was detected. In accordance with these findings, two patients were diagnosed with GBS and one patient was diagnosed with ICU neuropathy. EMG’s on five patients was found to be normal. In the review conducted by Koralnik et al., similar relationships between COVID-19 were reported^4^. In Koralnik et al.’s study, the EMG results were consistent with demyelination in two patients and axonal neuropathy in three patients. SARS-CoV-2 was not detected in CSF by means of RT-PCR in any of the patients^4^.

Despite the increasing number of case series, it is not clear whether SARS-CoV-2 causes GBS or whether this association is coincidental. Keddie et al.^19^ examined the relationship between COVID-19 disease and GBS, in their epidemiological cohort study they reported that there was no increase in the incidence of GBS during the COVID-19 pandemic; rather, there was a decrease in comparison with previous years. In addition, they reported that they did not detect any molecular similarity in SARS-CoV-2 that could cause, such as molecular mimicry, as in *C. jejuni* and CMV^19^. It has been theorized that GBS may occur due to different unidentified autoantibodies, viral neurotoxins or, with low probability, direct infection of the peripheral nerve by the viruses^20^.

In our study, the most common reason for requesting consultations was delirium (61.7%). The mean age of the patients with delirium was significantly higher than the age of those without delirium (p<0.001). In addition, ferritin (p=0.04) and CRP levels (p=0.015) were found to be significantly higher and lymphocyte levels (p=0.048) were significantly lower in patients with delirium than in those without delirium. These findings support the notion that cytokine storms cause delirium, in these patients.

The death rate was statistically higher among patients with delirium. In fact, considering that the risk of developing delirium was proportional to the severity of the disease and advancing age, this finding was an expected result. On the other hand, there was no statistically significant difference between the patients with and without delirium, in terms of the duration of intubation and hospital stay. In the study conducted by Helms et al., increased levels of protein, Ig-G and IL-6 were detected in the CSF of patients hospitalized in the ICU due to COVID-19 who developed delirium, and SARS-CoV-2 RNA was not isolated. Therefore, like in our study, it was suggested that delirium was related to a systemic inflammatory response and cytokine storm^13^.

In the study conducted by the Strasbourg group, agitation was observed in 69% and confusion in 65% of the patients hospitalized in the ICU due to COVID-19^9^.

According to the results of this study, delirium (hyperactive or agitated, hypoactive and mixed-type) was the reason for requesting a consultation in more than half of the cases assessed. When the patients who required NC were evaluated according to the subtypes of delirium, it was found that hyperactive delirium was present in 75.8%, hypoactive delirium in 13.7% and mixed-type delirium in 10.5% of the delirium patients. There were no statistically significant differences between the subtypes of delirium in terms of RASS scores or respiratory, infectious or coagulation parameters. The presence of dementia and CRF caused a statistically significant increase in the rate of delirium development. Interestingly, the incidence of delirium was found to be statistically lower in patients with a history of CVD and acute CVD. We think that this was due to the fact that patients with CVD were not included in the delirium category, since the etiology of the changes of consciousness had been clarified.

The second most common reason for requesting a NC was acute focal neurological deficits (15.6%). In our study, the rate of patients with acute hemorrhagic CVD, among all the patients with COVID-19 pneumonia, was 0.25%. The D-dimer values ​​of patients with acute hemorrhagic CVD were found to be statistically significantly higher than those of patients without this (p=0.03). In this group, four of the six patients had been diagnosed with HT. Three of these patients were using low-molecular-weight heparin treatment prophylactically because of the previous diagnosis of COVID-19. Three patients were diagnosed with COVID-19 at the time of hemorrhagic CVD diagnosis. In this group, uncontrolled HT or prophylactic anticoagulant agents used for high D-dimer levels are likely to cause intracerebral hemorrhage. In a case series presented by Fayed et al., one of the three COVID-19 patients with intracerebral hemorrhage had an increased D-dimer level and one patient was using anticoagulant^21^.

In the present study, acute ischemic CVD patients constituted 1.1% of all hospitalized patients diagnosed with COVID-19 pneumonia. MRI revealed areas of acute infarction in the left hemisphere, at a rate of 16.7%; in the right hemisphere at a rate of 9.7%; and bilaterally at a rate of 4.2%. In a cohort study, ischemic stroke was reported in 2-6% of the patients hospitalized with COVID-19^3^. Although the rates of ischemic CVD in COVID-19 patients are variable, in our study we detected lower rates, compared with the literature. This situation may have been due to the fact that the anticoagulant and/or antiaggregant treatment was started as soon as the disease was detected, if there was no reason why this treatment would increase the risk of bleeding in the patients with COVID-19 pneumonia. According to our data, the mean thrombocyte level was found to be significantly higher in acute ischemic CVD patients than in those without this condition (p<0.001). The finding that thrombocytosis increased the risk of ischemia was expected. However, our findings contradict other studies in the literature, in which it was reported that high D-dimer, fibrinogen and CRP levels increased the risk of stroke, in patients with concurrent COVID-19 and acute ischemic CVD. In a cohort study, 96 stroke patients who experienced vascular events associated with proinflammatory coagulopathy were found to have high CRP, D-dimer and ferritin levels^3^. In addition, in a recently published study, Sonkaya et al. compared mid-cerebral artery blood flow velocities in 20 people diagnosed with COVID-19 and 20 healthy volunteers, by means of transcranial Doppler USG. They showed that cerebral autoregulation is impaired in COVID-19 patients. It was found that the mean blood flow velocities in patients diagnosed with COVID-19 were higher (p=0.00), and that vasomotor reactivity was decreased, compared with healthy volunteers (p=0.00). This situation was thought to have been due to endothelial dysfunction^22^.

The limitations of this study were that CSF examinations were not performed on any patient, CK values ​​were not included in the study and EEGs were not performed on the ICU patients presenting confusion.

In conclusion, we determined that NC were requested more frequently for intensive care patients. As expected, it was observed that the mean PaO_2_/FiO_2_ ratio and oxygen saturation levels of the patients hospitalized in the ICU were significantly lower than those of the patients in the ward. The most common neurological condition was delirium, and it was observed more frequently among critically ill patients. Contrary to data in the literature, the rate of acute ischemic CVD was low and there was a statistically significant positive correlation between thrombocytosis and acute ischemic disease. Also, contrary to expectations, there was a statistically significant relationship between elevated D-dimer levels and acute hemorrhagic CVD. We anticipate that the complications of SARS-CoV-2 seen in acute and long-term follow-ups will become more clearly defined for neurologists in the future. We think that further studies with larger patient groups are needed to support our data.
